# Anti-ICOSL New Antigen Receptor Domains Inhibit T Cell Proliferation and Reduce the Development of Inflammation in the Collagen-Induced Mouse Model of Rheumatoid Arthritis

**DOI:** 10.1155/2018/4089459

**Published:** 2018-10-17

**Authors:** Ronan O'Dwyer, Marina Kovaleva, Jiquan Zhang, John Steven, Emma Cummins, Deborah Luxenberg, Alfredo Darmanin-Sheehan, Miguel F. Carvalho, Matthew Whitters, Kenneth Saunders, Caroline J. Barelle

**Affiliations:** ^1^Immunocore, Long Wittenham, Oxfordshire OX14 4RY, UK; ^2^Elasmogen Ltd, Aberdeen AB25 2ZP, UK; ^3^Novartis Institutes for BioMedical Research (NIBR), Shanghai 201203, China; ^4^The Centre for Drug Research and Development (CDRD), Vancouver, BC, Canada V6T 1Z3; ^5^Pfizer, Cambridge Park Drive, Cambridge, MA 02140, USA; ^6^Pfizer; Global Biotherapeutics Technologies, Dublin D22, Ireland; ^7^UCB New Medicines, B1420 Braine-l'Alleud, Belgium

## Abstract

Lymphocyte costimulation plays a central role in immunology, inflammation, and immunotherapy. The inducible T cell costimulator (ICOS) is expressed on T cells following peptide: MHC engagement with CD28 costimulation. The interaction of ICOS with its sole ligand, the inducible T cell costimulatory ligand (ICOSL; also known as B7-related protein-1), triggers a number of key activities of T cells including differentiation and cytokine production. Suppression of T cell activation can be achieved by blocking this interaction and has been shown to be an effective means of ameliorating disease in models of autoimmunity. In this study, we isolated specific anti-ICOSL new antigen receptor domains from a synthetic phage display library and demonstrated their ability to block the ICOS/ICOSL interaction and inhibit T cell proliferation. Anti-mouse ICOSL domains, considered here as surrogates for the use of anti-human ICOSL domains in patient therapy, were tested for efficacy in a collagen-induced mouse model of rheumatoid arthritis where they significantly decreased the inflammation of joints and delayed and reduced overall disease progression and severity.

## 1. Introduction

Rheumatoid arthritis (RA) is a chronic, immune-mediated inflammatory joint disease affecting 0.5–1% of the global population and results in cartilage and bone damage as well as disability [1]. The root cause of this debilitating disease is unknown; however, increased understanding of the underlying pathology has resulted in the development of a number of effective drugs, typically with one of three modes of action: (i) neutralising the effects of inflammatory cytokines, (ii) T cell costimulation blockade, or (iii) B cell depletion. Currently approved biologic-based treatments for RA include TNF*α* antagonists. Three of the most successful are monoclonal antibodies targeting TNF*α* directly and blocking its binding to TNFRII: infliximab or remicade [2], adalimumab or Humira [3], and golimumab or Simponi [4]. Currently approved therapeutics also include an antibody Fab′ fragment conjugated to a polyethylene glycol (PEG) (certolizumab pegol or Cimzia) [5–7] and a fifth biologic, etanercept or Enbrel, which comprises of a fusion protein of TNFRII and the Fc region of human IgG1 [8]. Although targeting TNF*α* has been validated through proven therapeutic efficacy (and significant commercial value), not all patients respond with 25–40% of subjects failing to reach the desired ACR20 end point (20% improved response based on the American College of Rheumatology) during clinical trials [9–11]. These patients' outcomes, coupled with the fact that a follow-on study of patients on TNF*α* antagonist treatments showed that after 5 years only 44% were still taking their original therapy [12], are driving the current interest in alternative targets for treatment. To help those patients that exhibit poor or no response to TNF*α* blockade, there are a number of monoclonal antibodies seeking to treat or control the disease through an alternative biology (tocilizumab—a humanized anti-IL-6R IgG1; rituximab—a B cell-directed chimeric anti-CD20 IgG1; and abatacept—an anti-T cell costimulation inhibitor comprising an extracellular domain of CTLA-4 fused to IgG-Fc) [13–15]. However, despite these additional approaches, there still remains a significant proportion of patients that struggle to find a suitable, long-term therapy option.

Shark Ig novel antigen receptors (IgNAR) are naturally occurring binding proteins that play a pivotal role in the adaptive immune system of cartilaginous fish [16, 17]. Although there are structural similarities between IgNAR and mammalian antibodies and shared functional characteristics such as *in vivo* maturation, there is evidence to show that they are derived from a distinct evolutionary lineage [18]. Therefore, IgNAR could be considered a unique form of heavy chain-only antibody-like protein consisting of five constant domains followed by the variable domain (VNAR) which mediates antigen binding [19]. The lack of a light chain and therefore the lack of a corresponding hydrophobic VH-VL interface (seen in a conventional antibody) make VNARs small and highly soluble in water. Sequence analysis of VNARs has revealed a close relation to cell surface adhesion molecules and T cell receptors (TCR) further distinguishing them from classical antibodies [20–22]. Moreover, and unlike antibodies that generate a binding site composed of three regions of high sequence diversity (CDR1, CDR2, and CDR3) from both the variable heavy and light chain (6 in total), VNARs utilize four regions of diversity (CDR1, HV2, HV4, and CDR3) creating a 4-loop binding site within a single domain. The presence of additional noncanonical cysteine residues in frameworks 2 and 4 and CDR loops defines a series of related VNAR isotypes or structurally distinct families with diverse paratope topologies capable of binding more cryptic or hidden epitopes [23–25]. Together, their selectivity for target, biochemical properties, and small size (the smallest naturally occurring immunological-like binding domain in the vertebrate kingdom at 11 kDa) make VNARs attractive candidates for therapeutic drug and diagnostic development [26–29].

Here, we provide evidence of the therapeutic potency and potential of shark VNAR domains in collagen-induced arthritis (CIA) in mice. This model reflects many of the immunological, histological, and clinical hallmarks of RA in humans including synovitis and cartilage and bone erosion [30].

To differentiate from the existing portfolio of anti-RA TNF*α*-based therapeutics in the clinic, we chose to target and neutralise the activity of ICOSL. ICOSL, also known as B7-related protein (B7RP-1), CD275, and B7 homologue (B7h), is a cell surface antigen expressed constitutively on antigen-presenting cells (APCs) such as B cells, activated monocytes, and dendritic cells and is the ligand for the B7 family member, ICOS (CD278) [31–33]. Initially, it was believed that its action was restricted to the activation phase of T cells and T cell-dependent B cell responses [34–36], but in more recent studies, the interaction between ICOS and ICOSL has been shown to play a role in the downstream survival and expansion of T cells (effector and regulatory) and in germinal centre (GC) formation [37, 38]. As well as promoting T_FH_ development, Liu et al. have demonstrated the importance of ICOSL within the GC where it acts as a molecular linkage between GC T_FH_ and B cells resulting in positive selection of bone marrow plasma cell formation, thereby also confirming a role in the control of long-lived humoral immunity [39].

Importantly, the growing understanding of ICOSL biology has now been translated into its use as a viable therapeutic target. The completion of a successful phase I clinical study in SLE patients and phase II in Sjogren's syndrome patients (both conducted by Amgen Inc.) demonstrated efficacy of the human anti-ICOSL mAb, prezalumab [40, 41]. ICOSL and its importance in antibody-mediated disease have also been verified in several preclinical models of human disease including RA, SLE, and uveitis [32, 36, 42–46] as well as in other models of arthritis (proteoglycan-induced arthritis (PGIA) and glucose-6-phosphate isomerase- (G6PI) induced arthritis), exemplifying the utility of anti-ICOSL-binding domains in the treatment of this immune disorder [44, 47, 48]. We have previously isolated VNAR domains from an immunised *Ginglymostoma cirratum* (nurse shark) library, which block the ICOS/ICOSL interaction, and went on to demonstrate their efficacy in a mouse model of noninfectious uveitis [42]. Here, we have selected and ranked domains isolated from a synthetic VNAR library. This library is based on optimized *Squalus acanthias* spiny dogfish frameworks and contains significant engineered binding loop diversity. We have demonstrated the efficacy of anti-ICOSL VNARs in a model of RA, extending their potential as therapeutics for treatment of a range of autoimmune diseases.

## 2. Materials and Methods

### 2.1. VNAR Phage Display Library Screening

All clones were isolated from a synthetic VNAR library containing 100 billion unique clones. Solid-phase, phage display library antigen selections were carried out as detailed previously [49] using MaxiSorp immunotubes (Nunc, 444474) coated with 1–0.1 *μ*g/ml antigen in PBS pH 7.4. Predecorated biotinylated antigen bead selection protocols were adopted from our previous work [42]. Outputs from each selection round were screened for antigen-specific binders by monoclonal phage and periplasmic extract ELISAs against human or mouse ICOSL and unrelated protein controls at 1 *μ*g/ml in PBS coating concentration. Phage binders were detected using HRP-conjugated anti-M13 antibody (GE Healthcare, 27942101), and periplasmic protein was detected using HRP-conjugated to an anti-c-Myc antibody (Roche, 118 141 50 001).

### 2.2. Expression and Purification of VNAR Fc-Fusion Proteins

Selected positive monomeric VNAR domains were PCR-amplified and subcloned into a proprietary Fc-fusion mammalian expression vector. Proteins were transiently expressed in HEK 293 cells and subsequently purified by Protein A-Sepharose. Expression levels of VNAR-Fc fusion proteins were generally in the region of 50–60 mg per litre using serum-free media. Postexpression cells and debris were removed from conditioned media by centrifugation and 0.2 *μ*m filtration. Following affinity chromatography, as detailed above, proteins were subjected to a final polishing step by size-exclusion chromatography (SEC) using a Superdex 200 26/60 column equilibrated with PBS. Eluted peaks from SEC were concentrated using Amicon ultrafiltration units and protein concentrations determined by UV spectroscopy. Electrophoresis of purified protein samples was performed on NuPAGE 4–12% Bis-Tris gels using the MOPS buffer system (Invitrogen) in accordance with the manufacturer's instructions. Proteins were then visualised by silver staining (Life Sciences, SilverQuest LC6070) and purity determined prior to *in vivo* experimental work.

### 2.3. Cell-Based Binding Assays

CHO cells expressing human or murine ICOSL were grown to confluency in DMEM/F12+ 5% FBS media, in 96-well cell culture plates (Greiner, Bio-One). Anti-ICOSL-VNAR-Fc (50 *μ*l) was added to the corresponding cells. Cells were incubated for 1 h at 16°C, gently washed 3x with DMEM/F12+ 2% FBS, and incubated for another 40 min at 16°C with goat anti-human Fc-HRP (SIGMA) diluted 1 : 10000 in the same media. Cells were washed a further 3 times with DMEM/F12+ 2% FBS media and once with PBS; lastly, TMB substrate was added and allowed to develop.

### 2.4. Cell-Based Ligand-Receptor Blocking Assays

CHO cells expressing murine or human ICOS receptor were used in blocking assays as described in our previous publication [42].

### 2.5. Murine D10 T Cell Proliferation Assays

Tosyl-activated magnetic Dynabeads were coated per product insert instruction with mICOSL, anti-mu CD3e, and hIgG1 filler (1 *μ*g ICOSL/0.5 *μ*g anti-CD3/3.5 *μ*g hIgG1 per 1 × 10^7^ beads). Prior to assay setup, beads were titred to determine the optimal concentration to give a reading of approximately 8000–40,000 CPM. Beads (50 *μ*l/well) were added to a 96-well plate containing titred antibody diluted in 100 *μ*l of RPMI, 10% FCS, 2 mM glutamine, penicillin/streptomycin, 10 mM HEPES, 1 mM Na pyruvate, 2 g/l glucose, and 50 *μ*M BME. D10.G4.1 cells were washed 4x with assay media and resuspended in the above medium plus 10% rat T stimulatory factor with Con A (BD cat#354115), 2.5 ng/ml IL-2, and 10 pg/ml IL-1 alpha to 8 × 10^5^ cells/ml and added at 50 *μ*l/well = 40,000 cells/well. All wells were brought up to a final volume of 200 *μ*l and incubated for 48 h. ^3^H-thymidine (1 *μ*Ci/well) was added and incubated for 5–7 h. Cells were then harvested and counts taken. T cell proliferation assays to assess anti-hICOSL VNAR domains were conducted using primary human T cells isolated from normal healthy donors. The method, in brief, was as follows: for the primary plate coating, 1 *μ*g/ml anti-huCD3 clone OKT3 (eBioscience cat. #16-5889aCD3) plus 10 *μ*g/ml anti-hIgG (Jackson ImmunoResearch cat. #109-006-098) in PBS in a total of 100 *μ*l/well was added to a 96-well plate and incubated overnight at 4°C. Residual solution was removed and the plate washed twice with PBS. For the secondary coating, 4 *μ*g/ml rhB7-2/CD86-Fc (R&D Systems cat. #141-B2-100) plus 500 ng/ml rhB7-H2-Fc (R&D Systems cat. #165-B7-100) was added in PBS at 100 *μ*l/well and incubated for 3 h at room temperature followed by washes with PBS. Media (50 *μ*l) were added to all wells of the assay plate in addition to 50 *μ*l CD4^+^ T cells (diluted to give 2 × 10^6^ cells/ml) and 50 *μ*l of test antibodies diluted to 3x, with the desired final concentration in media giving a final cell concentration of 1 × 10^5^ cells/well. The samples were incubated for 3 days then pulsed with 1 *μ*Ci/well of ^3^H-thymidine for 6–8 h on day 3 and counts measured.

### 2.6. FACS Analysis

Parental and mICOSL and hICOSL ligand-expressing CHO cells were washed in PBS and removed from flasks by the addition of PBS and 5% EDTA at 37°C for 10–15 min. Cells were monodispersed by pipetting up and down against the surface of the flask, spun down at 1200 rpm, and resuspended in DMEM plus 5% FCS. Cells were aliquoted at a density of 0.5–1 × 10^6^ cells/well into a 96-well U-bottomed plate. Cells were incubated with 100 *μ*l tissue culture supernatant containing HEK293 VNAR-Fc expressed proteins for 30 min at 16°C followed by 3x washes with PBS plus 2% FCS. Cells were then incubated with 100 *μ*l anti-hFc biotin (eBioscience #13-4998) at 1 *μ*g/ml for 30 min at 16°C. After 3x washes with PBS plus 2% FCS, streptavidin APC (eBioscience #17-4717) was added at 1 *μ*g/ml for 30 min at 16°C. After 1x wash with PBS plus 2% FCS, cells were resuspended in 400 *μ*l PBS plus 2% FCS and transferred into FACS tubes for analyses on a FACSCanto 2.

### 2.7. PK/PD Study

Test protein (CC3-Fc) was assessed for endotoxin, viral, mycoplasma, and bacterial contamination to ensure quality standards required for animal testing were achieved. A group of six DBA1 mice were injected with 4 mg/kg of CC3-Fc either intravenously or subcutaneously. Blood samples (0.3 ml) were collected after 1, 3, 6, 24, 48, 120, 168, 336, and 340 hours via the saphenous vein into EDTA tubes following a spin at 3000 × g, 4°C for 10 min for plasma recovery.

### 2.8. Plasma Pool down and LC-MS Analyses of PK Samples

CC3-Fc concentrations in plasma were analysed by quantitative LC-MC method described by Steven et al. [50]. Briefly, 50 *μ*l Protein A-Sepharose was added to wells of a Millipore filter plate and conditioned with PBS pH 7.4. Plasma samples were diluted 1 : 4 with PBS and added to the filter plate, incubated at room temperature and agitated at 700 rpm for 1 h. Wells were washed four times with 200 *μ*l PBS using a vacuum manifold. Bound Fc protein was eluted twice with 25 *μ*l of 100 nM glycine pH 2.0 into a low-bind deep-well block and neutralised by adding 7.5 *μ*l 2M Tris pH 8.0. Eluted protein samples were then trypsin-digested by addition of 20 *μ*l of proteomics-grade trypsin (made up at 20 *μ*g/mL in 10 mM CaCl_2_, 50 mM Tris-HCl, pH 8.0) followed by incubation at 37°C for 18 h prior to LC-MS/MS analysis. Samples were analysed using LC-MS/MS by monitoring tryptic signature peptides resulting from CC3-Fc present in the samples.

### 2.9. Murine Collagen-Induced Arthritis Model

All animal work was conducted by Charles River Laboratories, Ann Arbor, Michigan. All animal studies were carried out under the Animals (Scientific Procedures) Act 1986 regulations (Home Office UK). Test proteins were assessed to ensure quality standards required for animal testing were achieved (as above). *In vivo* efficacy of anti-mICOSL hits were determined in a mouse model of rheumatoid arthritis (RA) based on Iwai et al. [51], where groups of 10 female DBA1 mice were injected with bovine collagen in Freund's Complete adjuvant (day 0) followed by a boost on day 20. Anti-mICOSL VNAR-Fc domains and positive (HK5.3 mAb) and negative controls (2V-Fc and rat CHOCK IgG2) were dosed intraperitoneal on days 19, 21, 23, and 25 at 15 mg/kg in PBS. Clinical scores and weight were measured twice weekly. Clinical scores were based on calliper measurements of footpad and digit inflammation: 1 pt/digit, 5 pts/swollen footpad, and 5 pts/swollen ankle therefore giving a possible total of 15 pts/foot and 60 pts/animal. Histology was conducted on the back left foot of each animal where sections of 100 *μ*m steps were taken and stained with haematoxylin & eosin and scored for inflammation, pannus formation, cartilage damage, bone resorption, and periosteal change.

### 2.10. Statistical Analysis

Significant differences between experimental groups were analysed by the Mann-Whitney *U* test. Values of *p* = 0.05 were considered to be significant.

## 3. Results

### 3.1. Isolation of ICOSL-Specific VNAR Domains

**Figure 1:**
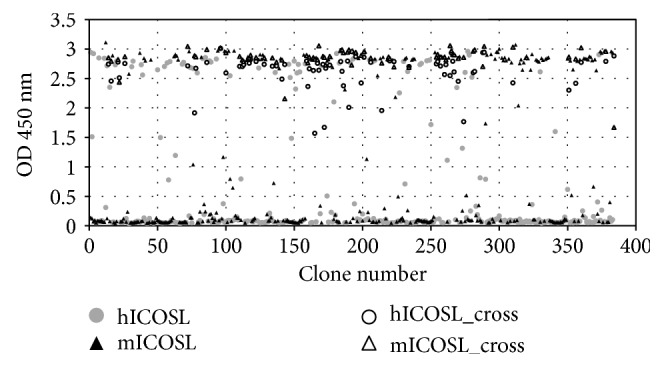
Selection of ICOSL-specific VNAR domain outputs from selection against mICOSL or hICOSL tested for binding to both ligands in ELISA. Each triangle and circle denote a clone; black fill triangles are clones binding to mICOSL only, and grey fill circles are clones binding to hICOSL only; open circles are cross-reactive clones binding to hICOSL, and open triangles are the same clones binding to mICOSL.

ICOSL-specific domains were isolated from a synthetic VNAR library using phage display technology. Three selection rounds were sufficient to obtain panels of VNARs specific to mouse ICOSL, human ICOSL, or cross-reactive clones (Figure 1). We have previously reported the isolation of anti-mouse ICOSL binders from a *Ginglymostoma cirratum*-immunised library which were cross-reactive with human and mouse ICOSL. Although these binders could recognise both species forms, they could only block mouse ligand-receptor interactions and not the interaction between human ligand and its receptor [42]. In an attempt to bias the selection of VNARs from the synthetic library that showed both receptor-ligand blocking and species cross-reactivity, a cell-based blocking assay as well as a cross-reactive selection campaign were introduced at an early stage of screening, with parallel selection and screening for mouse only or human only targets also included. In addition, two different antigen presentation regimes were utilized, with biotinylated antigen immobilised on streptavidin-coated beads and direct immobilisation of antigen on immunotubes, both conducted to explore the full diversity of the library. A total of 24 unique anti-murine ICOSL VNAR clones which block the mICOS/mICOSL interaction and 12 unique anti-human ICOSL blocking the hICOS/hICOSL interaction were identified. In this instance, and despite the stringency of the selection conditions, selected cross-reactive clones were again not able to block either murine or human receptor/ligand interactions (data not shown).

To determine the efficacy of VNAR domains in an *in vivo* mouse model, all selected VNAR clones were converted into an Fc-fusion format to facilitate an extension of serum half-life and binding avidity. VNAR domains alone are cleared rapidly *in vivo*, and whilst this may be useful for some therapeutic applications, an extended half-life was expected to be required for full efficacy [17]. VNAR-Fc proteins were expressed transiently in HEK-293 cells and purified by protein A affinity chromatography followed by size exclusion chromatography. All converted clones expressed protein at levels of 50–60 *μ*g/ml.

### 3.2. VNAR Domains Assessed by In Vitro Cell-Based Binding Assays

**Figure 2:**
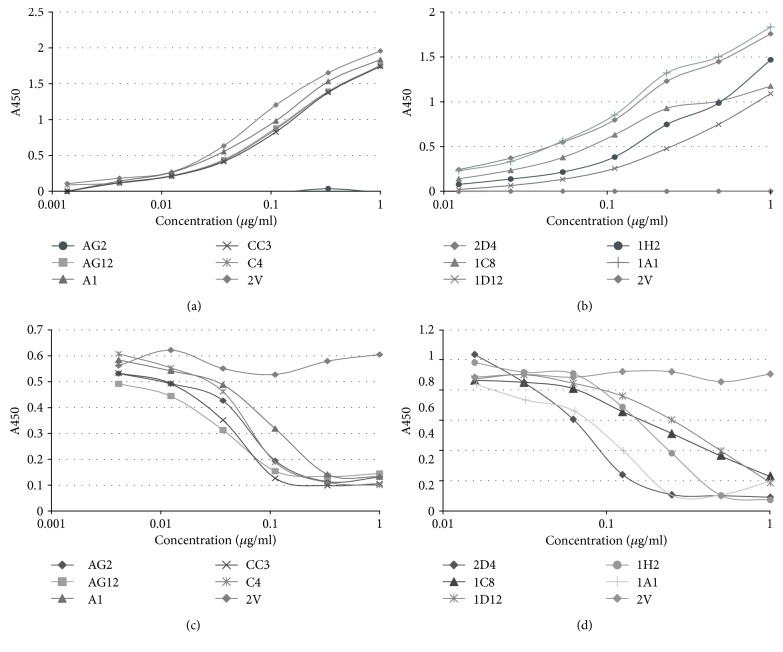
Cell-based binding and ICOS/ICOSL blocking assays. Lead anti-mICOSL and anti-hICOSL VNAR-Fc proteins were tested for binding to the CHO cell surface expressed ICOSL and their corresponding efficacy in an ICOS/ICOSL blocking assay. (a) Titration curves of anti-mICOSL VNAR-Fc domains binding to mICOSL-CHO cells. (b) Titration curves of anti-hICOSL VNAR-Fc domains binding to hICOSL-CHO cells. (c) Concentration-dependent inhibition of mICOSL-Fc binding to cell surface expressing mICOS by the addition of serial dilutions of anti-mICOSL VNAR-Fc domains. (d) Concentration-dependent inhibition of hICOSL-Fc binding to cell surface expressing hICOS by the addition of serial dilutions of anti-hICOSL VNAR-Fc domains. 2V-Fc is the VNAR isotype control in each experiment.

The ability of purified anti-ICOSL VNAR-Fc domains to retain their ability to bind to cell surface expressing ICOSL ligand was confirmed by titration in cell-based ELISAs. Of the 24 anti-mICOSL VNAR-Fc proteins assessed, domains AG2, AG12, A1, CC3, and C4 were the most potent with IC_50_ values ranging from 0.15 to 0.35 nM. Consequently, these were selected as the lead anti-mICOSL VNARs based on both their cell binding (Figures 2(a)) and potent inhibition of the interaction between ICOSL and ICOS (Figure 2(c)). Similarly, the 12 selected anti-hICOSL clones were ranked based on binding (Figure 2(b)) and potency of neutralisation (Figure 2(d))—of these, 2D4, 1A1, 1C8, 1H2, and 1D12 (IC_50_ values ranging from 0.5 to 9.9 nM) were selected as leads. The VNAR domain 2 V was included as an isotype control for all *in vitro* and *in vivo* assays. This clone, originally isolated from the dogfish *Squalus acanthias*, was part of a sequence analysis of naïve VNARs from this species and has no known target, making it an ideal negative control for these and other published studies [17].

**Figure 3:**
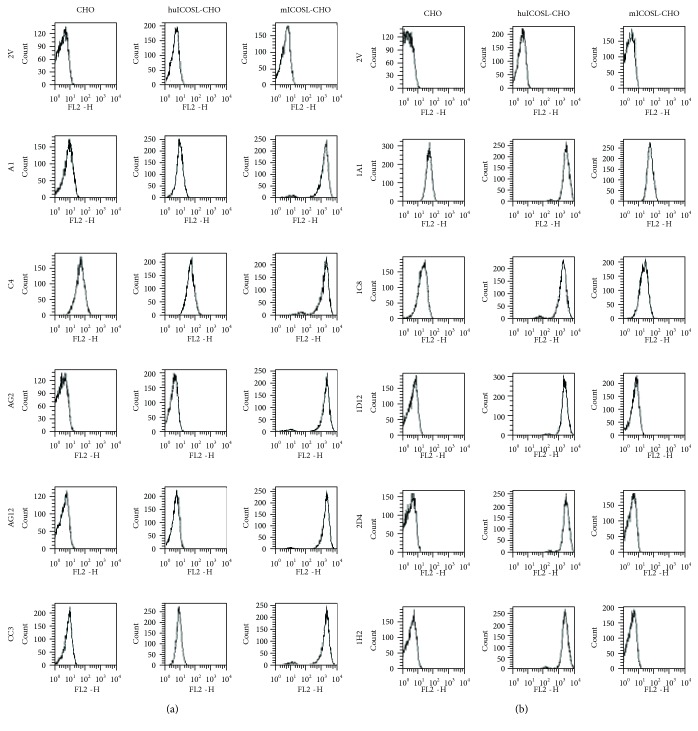
Flow cytometry analysis of lead domains binding to CHO cells. Lead anti-mICOSL and anti-hICOSL VNAR domains were tested for binding to cell surface expressing hICOSL, mICOSL, and parental CHO cells. (a) Anti-mICOSL VNAR domains were incubated with different CHO cell populations overexpressing either human or mouse ICOSL. (b) Anti-hICOSL VNAR domains were incubated with different CHO cell populations overexpressing either human or mouse ICOSL. To assess any nonspecific binding events, parental CHO cells were included as a cell control and 2V-Fc as a nonbinding VNAR domain control. Assays were repeated 3 times with representative data shown.

Cell surface target selectivity was assessed by flow cytometry analysis with CHO cells overexpressing human ICOSL or murine ICOSL. All of the anti-mICOSL domains showed clear binding to mICOSL-CHO cells and negligible binding to hICOSL-CHO compared to the parental CHO control, with the exception of clone C4 that showed a modest level of binding to hICOSL-CHO and control cells (Figure 3(a)). A similar range of specificities was seen for the anti-hICOSL domains. They all showed strong binding to hICOSL-CHO cells with clones 1A1 and 1C8, also displaying weak binding to control CHO cells (Figure 3(b)).

### 3.3. Suppression of T Cell Proliferation by Anti-ICOSL VNAR Domains

**Figure 4:**
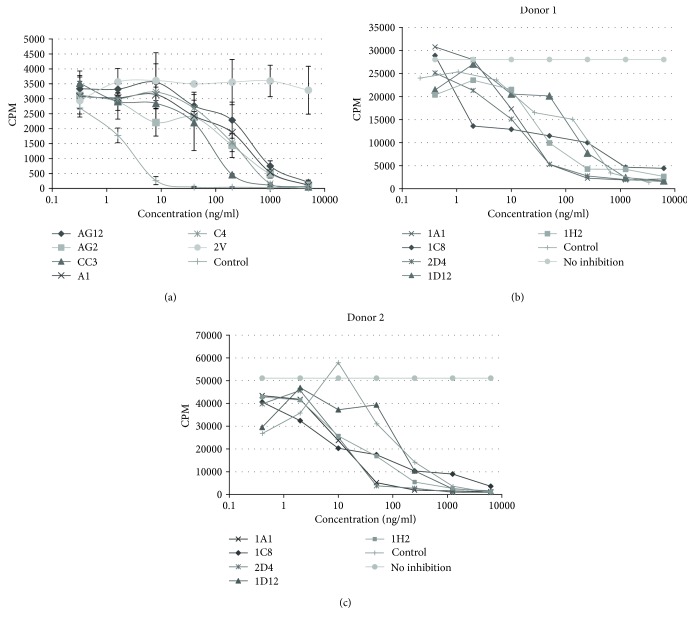
T cell proliferation assays mouse T cell line (a) and human primary T cell (b and c) proliferation assays were conducted to determine the efficacy of anti-mICOSL and anti-hICOSL VNAR-Fc proteins to block ICOS-ICOSL-induced cell proliferation, respectively. (a) Concentration-dependent inhibition of proliferation by lead anti-mICOSL VNAR-Fc proteins (*n* = 3). 2V-Fc was included in each experiment as the VNAR isotype control. (b, c) Efficacy of lead anti-hICOSL VNAR-Fc domains in two independent primary human T cell proliferation assays from two different donors. The positive controls in each experiment were in-house developed anti-human and mouse ICOSL monoclonal antibodies. Assays were repeated 3 times with representative data shown.

ICOSL, on the surface of antigen-expressing cells, plays an integral role in the activation of CD4^+^ T cells through its interaction with ICOS. This cell-cell interaction results in a cascaded proliferation of helper T cells. To recreate this effect *in vitro* and determine whether the identified lead domains could block this downstream intracellular signalling, T cell assays were performed where murine or primary human T cells were activated by the addition of ICOSL and costimulators. All five anti-mICOSL VNAR-Fc proteins inhibited T cell proliferation in a dose-dependent manner compared to the 2 V isotype control (Figure 4(a)). Domain CC3 consistently showed the greatest efficacy with an average EC_50_ of 792 pM ± 143 (*n* = 11). The ability of the anti-hICOSL VNAR domains to inhibit T cell proliferation was determined using primary cells isolated from individual donors (Figure 4(b)). All five lead domains demonstrated picomolar EC_50_ values in this assay with the best clones having values of 8.5 ± 3.6 pM (1A1), 9 ± 2 pM (1C8), and 7.5 ± 2.1 pM (2D4).

### 3.4. Pharmacokinetics, Dynamics, and Metabolism of Anti-mICOSL VNAR Domain

**Figure 5:**
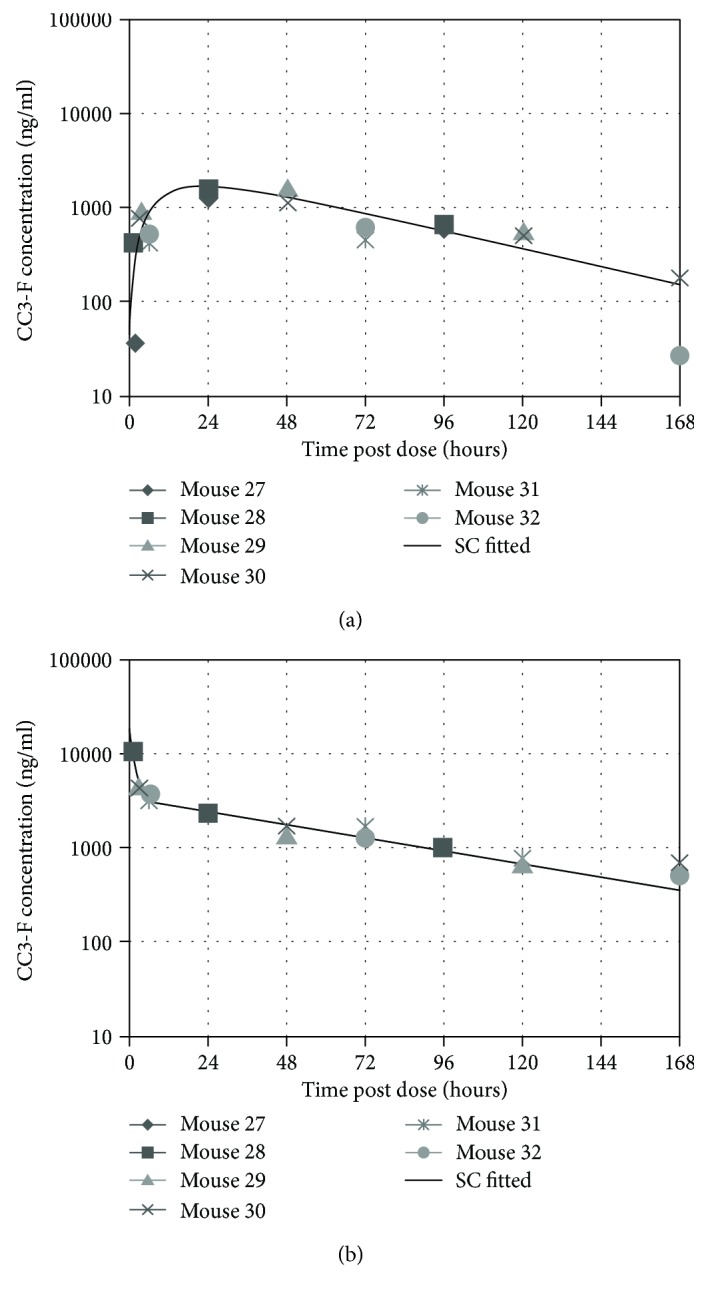
The PK profiles for an anti-mICOSL VNAR-Fc CC3-Fc dosed at 4 mg/kg i.v. and s.c. Composite profiles derived from 6 animals per route of administration: curve-fitted subcutaneously (a) and curve-fitted intravenously (b).

A preliminary PK/PD study was conducted within the mouse strain matched to the intended arthritis model, DBA1, with a single dose at 4 mg/kg of CC3-Fc administrated either intravenously (i.v.) or subcutaneously (s.c.). The Fc part of the CC3-Fc molecule was utilized for sample enrichment using the Protein A-Sepharose capture step, and protein concentrations were determined in plasma samples. A quantitative LC-MS/MS method previously described by Steven et al. [50] was used for analysis and PK assessment. The PK profiles are shown in Figure 5 and the pharmacodynamic parameters summarised in Table 1.

### 3.5. Evaluation of Anti-mICOSL VNAR Domains in a Murine Model of Collagen-Induced Arthritis (CIA)

**Figure 6:**
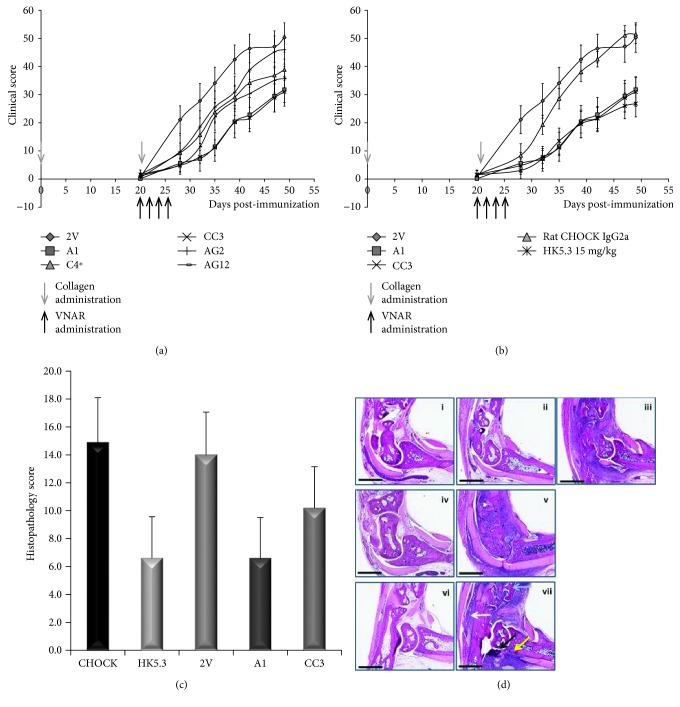
Clinical scores and histopathology sections from the CIA study CIA study results. (a, b) Average clinical scores for each experimental group of 10 animals measured twice weekly for the duration of study. A collagen boost was given at day 20 and dosing of test samples at 15 mg/kg at days 19, 21, 23, and 25. Data shown as an average clinical score ± SEM and comparisons were made using the Mann-Whitney test. (c) Analysis of the ankle RA scores from the left hind joint from each animal: the level of inflammation, pannus formation, cartilage damage, bone resorption, and periosteal change was measured, and each of these parameters was allocated a score from 1 to 5. The summarised histopathology scores were plotted. (d) Left hind paws from each experimental animal were sectioned and stained with H&E. (i) A1-Fc, (ii) CC3-Fc, (iii) 2V-Fc, (iv) HK5.3, (v) IgG2a, (vi) dexamethasone, and (vii) vehicle control. Black arrow: an example region of bone erosion; grey arrow: an example region of bone resorption; white arrow: cellular infiltrate; yellow arrow: pannus formation. The size bar represents 2 mm.

Protein homology between mouse and human ICOSL is approximately 43%; therefore, only the anti-mouse domains could be tested in this surrogate RA model. The CIA model was carried out by Charles River Laboratories, Ann Arbor, Michigan as follows. DBA/1J mice were immunised with bovine collagen in Freund's complete adjuvant (day 0) and then boosted with a second administration of collagen on day 21. Fc-fusion versions of anti-mICOSL VNAR, together with the isotype format control 2 V, were administered intraperitoneally (i.p.) at 15 mg/kg on days 19, 21, 23, and 25, except for clone C4 which was administrated at a concentration of 8.4 mg/kg. Clinical scores were measured twice weekly as described in the Materials and Methods. The average clinical score was measured in each experimental group (10 animals) over the 50-day time period of the study with a score of 60 representing the maximum level of inflammation (Figures 6(a) and 6(b)). Administration of A1 and CC3 VNAR-Fc proteins resulted in a significant decrease (*p* < 0.05) in overall clinical score and a clear lag in the onset of any disease compared to the relevant isotype control. The anti-murine ICOSL monoclonal IgG HK5.3 was used as the positive biologic control [51]. The calliper measurements of footpad and digit inflammation (clinical score) were similar for A1, CC3, and the positive control (HK5.3) groups. The reduction in inflammation translated into visible histopathology differences at the tissue level in the left hind limb of each animal. Animals treated with dexamethasone, a potent anti-inflammatory corticosteroid, presented with no apparent disease pathology (vi) compared to the vehicle control (vii) which exhibited extensive cartilage destruction, bone erosion, pannus formation, and granulocyte infiltration (Figure 6(d)). These hallmarks of arthritis were also evident in both isotype control groups (iii and v) whereas A1- (i) and CC3- (ii) treated animals show greatly reduced joint damage similar to the levels seen in HK5.3-treated samples (iv). Based on the histopathology sections, an analysis of the ankle RA scores from the left hind joint from each animal was conducted by measuring the level of inflammation, pannus formation, cartilage damage, bone resorption, and periosteal change. Each of these parameters was allocated a score from 1 to 5 and the summarised histopathology scores plotted in Figure 6(c).

## 4. Discussion

According to the latest population studies of people aged 18 to 64, about one in three (both men and women) will have doctor-diagnosed arthritis and/or report joint symptoms consistent with a diagnosis of arthritis [52]. In the last decade, our knowledge of the underlying pathobiology of rheumatoid arthritis has significantly increased with targeted biological therapies providing clear evidence that multiple immunological and inflammatory pathways operate. Each year, new roles for cytokines, mediators, and pathways that show additional promise in unravelling the full complexity of the pathways driving RA disease are published [53–55]. ICOSL induces important costimulatory signals delivered through ICOS receptor molecules on the surface of the T cell, resulting in T cell activation. Therefore, it follows that interruption or blocking of this costimulatory signal pathway may provide a potential biological target and therapy option to treat RA.

Anti-ICOSL VNARs, derived from a synthetic *Squalus acanthias* library, were ranked based on their ability to inhibit the ICOS-ICOSL interaction. Five lead candidates were selected, and two of them showed comparable efficacy in a murine CIA model resulting in delayed onset of disease and reduced overall disease burden.

An important step in the development of biologics is the study of preclinical efficacy usually using rodent models. In some cases, a candidate molecule recognises and neutralises both the human and the orthologous protein in rat or mouse. For some targets, however, low species homology precludes the use of the same candidates in rodent models of preclinical efficacy with a second panel of rodent-specific binders used as surrogates to predict efficacy in a human setting [56–60]. We have tried previously and unfortunately failed to select neutralising species cross-reactive anti-ICOSL VNAR derived from an immunised shark library. Here, using a synthetic VNAR library, the introduction of cross-reactivity and blocking assays into the screening regime again generated VNAR domains capable of binding to both human and murine targets, but lacking the ability to block ICOS/ICOSL in both species. Therefore, the *in vivo* activity of anti-mICOSL-specific VNAR domains must again be considered as surrogates for the use of anti-hICOSL VNARs in patient therapy. It is also worth noting that in T cell proliferation assays, the selected anti-hICOSL VNAR domains demonstrate 100x higher levels of potency (EC_50_) than lead anti-mICOSL domains do, reinforcing the expectation that they would show excellent *in vivo* efficacy.

In the mouse CIA model HK5.3, a control rat anti-mouse ICOSL mAb, known to inhibit both Th1 and Th2 immune responses and ameliorate inflammatory arthritis [51], was used. When compared to HK5.3, the reduction in clinical score with A1 and CC3 VNAR-Fc domains was equivalent and significantly better than the 2V-Fc isotype control. In histopathological analysis, A1 and CC3 VNAR-Fc reduced cartilage destruction, bone erosion, pannus formation, and granulocyte infiltration, to the same extent as HK5.3 (Figure 6(d)). CC3 was administrated at ~50% lower dose but still had comparable efficacy.

The flexible reformatting of VNAR domains was exemplified by the addition of a human Fc region to facilitate an increase in serum half-life and avidity of binding. Preliminary, single-dose, pharmacokinetic/pharmacodynamic studies administrated intravenously or subcutaneously resulted in considerable bioavailability and serum half-life for both routes of administration (Figure 5). At only 80 kDa, VNAR-Fc provides a valuable therapeutic format for systemic administration. A new VNAR structure known as a Quad-X™ delivers an Fc fusion and quadravalency (100 kDa) and has shown even greater potency (10x) than that of the equivalent bivalent Fc format, for an anti-TNF*α* VNAR domain [61]. It would be interesting to determine if a Quad-X™ version of candidate anti-ICOSL domains would see a similar uplift in potency.

## 5. Conclusion

Anti-ICOSL VNAR domains selected from a synthetic library have been shown to block receptor/ligand interaction *in vitro* and in cell-based assays as well as to inhibit human T cell proliferation with pM potency. When this *in vitro* bioassay potency is combined with the use of anti-mICOSL VNAR-Fc *in vivo* as surrogate drugs for accelerated development, these biologics exhibit excellent efficacy in a predictive mouse model of CIA demonstrating their ease of reformatting, simplicity of production, and potential to bring relief to the 40% of patients that do not respond to first-line anti-TNF therapies in RA and other debilitating autoimmune diseases.

## Figures and Tables

**Table 1:** 

Parameter	Intravenous dose	Subcutaneous dose
Clearance (ml/h/kg)	17.1	28.1
Half-life (h)	52	38
AUC (h^∗^*μ*g/ml)	234	142
Bioavailability		61%

## Data Availability

All data used to support the findings of this study are unavailable due to the commercially sensitive nature of the research.

## Abbreviations

ACR: American College of Rheumatology
APC: Antigen-presenting cells
CDR: Complementarity determining region
CIA: Collagen-induced arthritis
FW: Framework
GC: Germinal centre
HV: Hypervariable region
ICOSL: Induced costimulatory ligand
IgNAR: Immunoglobulin-like new antigen receptor
PEG: Polyethylene glycol
RA: Rheumatoid arthritis
SLE: Systemic lupus erythematosus
T_FH_: Follicular helper T cells
VNAR: Variable domain of shark new antigen receptor
